# Testing for a causal role of thyroid hormone measurements within the normal range on human metabolism and diseases: a systematic Mendelian randomization

**DOI:** 10.1016/j.ebiom.2024.105306

**Published:** 2024-08-26

**Authors:** Heba Alwan, Jian'an Luan, Alice Williamson, Julia Carrasco-Zanini, Isobel D. Stewart, Nicholas J. Wareham, Claudia Langenberg, Maik Pietzner

**Affiliations:** a MRC Epidemiology Unit, University of Cambridge School of Clinical Medicine, Institute of Metabolic Science, Cambridge, UK; b University of Bern, Institute of Primary Health Care (BIHAM), Bern, Switzerland; c University of Bern, Graduate School for Health Sciences, Bern, Switzerland; d Precision Healthcare University Research Institute, Queen Mary University of London, London, UK; e Computational Medicine, Berlin Institute of Health at Charité - Universitätsmedizin Berlin, Berlin, Germany

**Keywords:** Thyroid function, Mendelian randomisation, Health outcomes

## Abstract

**Background:**

Variation in thyroid function parameters within the normal range has been observationally associated with adverse health outcomes. Whether those associations reflect causal effects is largely unknown.

**Methods:**

We systematically tested associations between genetic differences in thyrotropin (TSH) and free thyroxine (FT4) within the normal range and more than 1100 diseases and more than 6000 molecular traits (metabolites and proteins) in three large population-based cohorts. This was performed by combining individual and summary level genetic data and using polygenic scores and Mendelian randomization (MR) methods. We performed a phenome-wide MR study in the OpenGWAS database covering thousands of complex phenotypes and diseases.

**Findings:**

Genetically predicted TSH or FT4 levels within the normal range were predominately associated with thyroid-related outcomes, like goitre. The few extra-thyroidal outcomes that were found to be associated with genetic liability towards high but normal TSH levels included atrial fibrillation (odds ratio = 0.92, p-value = 2.13 × 10^−3^), thyroid cancer (odds ratio = 0.57, p-value = 2.97 × 10^−4^), and specific biomarkers, such as sex hormone binding globulin (β = −0.046, p-value = 1.33 × 10^−6^) and total cholesterol (β = 0.027, p-value = 5.80 × 10^−3^).

**Interpretation:**

In contrast to previous studies that have described the association with thyroid hormone levels and disease outcomes, our genetic approach finds little evidence of an association between genetic differences in thyroid function within the normal range and non-thyroidal phenotypes. The association described in previous studies may be explained by reverse causation and confounding.

**Funding:**

This research was funded by the 10.13039/501100001711Swiss National Science Foundation (P1BEP3_200041). The Fenland study (DOI 10.22025/2017.10.101.00001) is funded by the 10.13039/501100000265Medical Research Council (MC_UU_12015/1, MC_PC_13046 and MC_UU_00006/1). The EPIC-Norfolk study (DOI 10.22025/2019.10.105.00004) has received funding from the 10.13039/501100000265Medical Research Council (MR/N003284/1, MC-UU_12015/1, MC_PC_13048 and MC_UU_00006/1).

**Research in context**

**Evidence before this study**

Several observational studies have pointed towards an association between thyroid function within the normal range and adverse health outcomes, although results are conflicting. In addition, few studies have attempted to systematically assess whether multiple health outcomes are casually associated with thyroid function using Mendelian randomization.

**Added value of this study**

We assessed whether genetic differences in thyroid function within the normal range are causally associated with a wide range of health outcomes and molecular traits using Mendelian randomization. We found that genetically predicted thyroid hormone levels within the normal range were mainly associated with thyroid-related outcomes, and very few extra-thyroidal outcomes.

**Implications of all the available evidence**

Our results suggest that findings from previous observational studies linking thyroid function within the normal range and a variety of adverse health outcomes may be explained by reverse causation or confounding. These findings may indicate that screening individuals with thyroid function at the upper or lower limit of normal for diseases may only be beneficial for a small number of non-thyroidal outcomes, if at all.

## Introduction

Overt and subclinical thyroid dysfunction have been shown to be observationally associated with multiple diseases.^1^ More recently, observational studies have extended some of these associations as well as novel ones to variations of thyroid function parameters even within population-reference ranges, including non-thyroidal phenotypes such as atrial fibrillation, stroke, cardiovascular disease, and fractures,^1^, ^2^, ^3^, ^4^, ^5^ among many others. This may be explained by a combination of two facts. Firstly, thyroid hormone receptors are expressed in virtually all organs in the body^6^ and hence alteration in thyroid hormone signalling may affect multiple organ systems as seen in patients with rare loss-of-function mutations.^7^ Secondly, it is believed that each individual has a unique euthyroid set point controlling the hypothalamus–pituitary–thyroid axis,^8^^,^^9^ that is, similar plasma concentrations of TSH may lead to different FT4 concentrations in individuals. This concept is supported by previous studies demonstrating that thyroid hormone concentrations are maintained within fairly narrow ranges.^10^^,^^11^ However, large variations exist in thyroid hormone concentrations among individuals in the population.^10^ Therefore, a change in the TSH value in one individual that is outside their personal reference range, but still within the population reference range, might already indicate subtle changes with possible systemic consequences.^10^^,^^11^

Such systemic consequences have been suggested by a number of observational studies linking variation in thyroid function parameters to small molecules and proteins in blood or urine, potentially suggesting pathways that may eventually lead to different diseases.^12^^,^^13^ If these observational associations were proven to be causal, monitoring of thyroid function even among asymptomatic individuals might be justified to reduce the associated disease burden.

Results from observational studies that describe the association between biomarkers and disease outcomes are often limited by confounding and reverse causation.^14^ Genetic epidemiological methods, such as Mendelian randomization (MR), can be used to test for evidence of causality as these approaches are not affected by confounding or reverse causation. MR uses genetic variants as instrumental variables for testing the causal effect of the exposure (e.g., thyroid function) on the outcome.^14^ For example, MR studies instrumenting genetically predicted thyrotropin (TSH) and free thyroxine (FT4) levels provided evidence of their role in atrial fibrillation,^15^^,^^16^ dyslipidemia,^17^ and pulse pressure^18^ supporting findings from clinical observations and functional studies. Conversely, MR analyses did not provide support for causality between thyroid function and other cardiovascular diseases, including stroke.^16^ However, most MR studies to date have focused on one or a limited number of outcomes, and systematic assessments, that acknowledge the ubiquitous expression of thyroid hormone receptors and a personal set-point of the hypothalamic–pituitary–thyroid axis,^9^ are lacking.

Clarifying whether the association between thyroid function within the normal range and certain health outcomes is causal can have clinical implications. If the association is shown to be causal, individuals with a thyroid function at the upper or lower limit of the population normal range may benefit from careful monitoring of their thyroid function and/or screening for associated adverse outcomes. We therefore studied more than 1100 diseases and more than 6000 molecular traits to contextualize potential health consequences of the variation in thyroid function within the normal range in a systematic manner.

## Methods

### Instrument selection and genotyping

Single nucleotide polymorphisms (SNPs) associated with thyroid function were identified from the largest genome-wide association study (GWAS) meta-analysis for TSH (119,715 individuals)^19^ and the largest GWAS available for FT4 (72,167 individuals),^20^ explaining 13.3% and 4.8% of the total variance in TSH and FT4 levels, respectively.^19^^,^^20^ The F-statistic for each of the TSH SNP was more than 23.7^19^ and more than 10 for FT4 SNPs.^20^ There were, in total, 99 SNPs for TSH and 31 SNPs for FT4 (Supplementary Table S1) that passed genome-wide significance levels (p < 5 × 10^−8^). The GWAS meta-analysis for TSH used data from the Nord-Trøndelag Health Study (HUNT study), Michigan Genomics Initiative, and the ThyroidOmics consortium.^19^ In the HUNT study, individuals with self-reported thyroid disorders or blood tests indicating overt hypothyroidism were excluded.^19^ In the Michigan Genomics Initiative, individuals with thyroid disorders (thyroid cancer, hypothyroidism, thyroiditis, or other disorders of the thyroid) were excluded.^19^ In the ThyroidOmics consortium, only normal range-TSH levels were included in the analyses.^19^ SNPs associated with FT4 were derived from the ThyroidOmics consortium, where only SNPs associated with normal-range FT4 levels were included in the analyses.^20^

A genetic risk score (GRS) was constructed for both TSH and FT4 using a weighed sum of the number of risk alleles within each of the three above-mentioned studies. Briefly, a GRS aggregates the effect of a large number of alleles to estimate the risk of a trait in one individual.^21^^,^^22^ Only data from unrelated participants of European ancestry were included (n = 9902 for the serum metabolite analysis in the EPIC-Norfolk study, n = 8350 for the serum protein analysis in the Fenland Study, n = 351,934 for the UKBB biomarker analysis, n = 84,366 for the UK Biobank (UKB) NMR biomarker analysis, n = 351,934 for the UKB PheWAS). For the TSH GRS, one SNP was not available in the UKB and the EPIC-Norfolk study (rs121908872) and two were not available in the Fenland study (rs121908872 and rs139352934). We omitted SNPs that have previously been shown to have pleiotropic effects (e.g., ABO locus).^23^

#### Study cohorts

##### EPIC-norfolk study

To explore associations between the GRS for TSH and FT4 and plasma metabolites, we used data from the EPIC-Norfolk study.^24^ The EPIC-Norfolk study is a cohort of 25,639 middle-aged individuals from the general population of the county of Norfolk in the east of England. Metabolomic profiling was performed into two sub-cohorts of 5989 and 5977 participants quasi-randomly selected from the full cohort after exclusion of a type 2 diabetes case cohort.^23^ Details on the measurement of metabolites and the methods involved have been previously described.^23^^,^^25^ Briefly, 1014 metabolites were measured in the non-fasting state. The measured metabolites include a wide range of molecules, from products of human metabolism such as lipids and amino acids to substances of exogenous origin.^23^ Metabolites were measured by four methods using a Waters ACQUITY ultra-performance liquid chromatography and a Thermo Fisher Scientific Q Exactive high-resolution/accurate mass spectrometer.^23^ Metabolite levels were log-transformed, winsorized (the tails of the distribution were defined as the mean ± 5 × standard deviation), and then standardized. Participants were genotyped using the Affymetrix Axiom or Affymetrix SNP5.0 genotyping array.^26^

##### Fenland study

Associations between genetic determinants of thyroid function and plasma protein levels were explored using data from the Fenland cohort, a population-based cohort of 12,435 individuals from Cambridgeshire, England where 12,084 participants underwent proteomic profiling.^27^ Fasting plasma measurements of proteins were performed by SomaLogic Inc (Boulder, CO, USA) using an aptamer-based technology (SomaScan proteomic assay).^27^ The SomaScan assay uses short single-stranded DNA molecules that are modified to bind to protein targets.^23^^,^^28^ DNA microarrays are then utilized to determine the relative amount of aptamers binding to protein targets.^27^^,^^28^ Further details can be found elsewhere.^27^^,^^28^ Only aptamers that passed Somalogic quality control and were human proteins targets were included in the present analysis (4979 out of the 5284 aptamers). Detailed methods on SomaLogic normalization have been previously described.^28^ Briefly, to account for variation in hybridization within runs, hybridization control probes are used to generate a hybridization scale factor for each sample. A normalisation procedure (adaptive median normalisation (AMN)) was used to control for total signal differences between samples due to variation in overall protein concentration or technical factors such as reagent concentration, pipetting or assay timing.^29^ This ensures quantitative comparability between sample sets. For AMN, a ratio between each aptamer's measured value and a reference value from an external reference population is calculated.^29^ The median of the ratios is calculated for each of the three dilution sets (20%, 0.5% and 0.005%) and then applied to each dilution set to shift the intrapersonal distribution of protein intensities to match the reference population.^29^ Before conducting statistical analysis, protein data were log-transformed and standardized. Genotypes were measured using the Affymetrix UK Biobank Axiom array, Illumina Infinium Core Exome 24v1, and Affymetrix SNP5.0.^27^

##### UK biobank

The UKB is a population-based study including around 500,000 individuals aged 40–69 years at the time of recruitment (between 2006 and 2010) in the UK.^30^^,^^31^ A wide range of biological and phenotypic data were collected for each participant.^30^^,^^31^ The analyses performed in this paper were conducted under UK biobank application number 44448.

#### UKB biochemistry marker and cardiovascular risk factors and ^1^H nuclear magnetic resonance (NMR) metabolomic biomarker data

Thirty-three biochemistry markers and cardiovascular risk factors were included in the analyses. All biochemistry markers were inverse rank normal transformed and some were additionally log-transformed (total triglycerides, lipoprotein A, oestradiol, testosterone, creatinine, C-reactive protein, cystatin C, alkaline phosphatase, alanine aminotransferase, aspartate aminotransferase, gamma-glutamyl transferase, total and direct bilirubin, lipoprotein (a), and rheumatoid factor). The development of high throughput ^1^H nuclear magnetic resonance (NMR) spectroscopy has additionally allowed the rapid measurement of a large number of metabolomic biomarkers.^32^ NMR metabolomic markers have currently been measured in approximately 120,000 participants in the UKB on the Nightingale platform. We used an R package to log-transform and remove technical variation in the measurement of the NMR metabolomic biomarker data, prior to performing the association analyses.^32^

To generate phecode-based outcome variables, we mapped ICD-9, ICD-10, Read version 2 and Clinical Terms Version 3 (CTV3) terms from self-report or medical health records, including cancer registry, death registry, hospitalizations (Hospital Episode Statistics), and primary care (subset, n = 214,667), to phecodes.^33^ We used any code that was recorded, irrespective if it contributed to the primary cause of death or hospital admission, to define phecodes. In total, 1570 phecodes were included in the analysis and we treated all participants with a respective code as a case, irrespective whether it occurred before or after the baseline examination. Participants in the UKB were genotyped using the Applied Biosystems UK BiLEVE Axiom Array by Affymetrix or the Applied Biosystems UK Biobank Axiom Array.^31^

##### Statistical analysis

The associations between the genetic scores and plasma metabolites were assessed using linear regression adjusted for age, sex, batch number, and the first ten genetic principal components. The associations between the genetic scores and plasma proteins were measured by linear regression adjusted for age, sex, test centre, and the first ten genetic principal components. Linear regression analysis adjusted for age, sex, test centre, batch, and the first ten genetic principal components was performed to evaluate the associations between the GRS for TSH and FT4 and biochemistry markers and NMR metabolomic biomarkers in the UKBB. To assess the associations between genetic differences in thyroid hormone levels and health outcomes in the UKB, we conducted a phenome-wide association study (PheWAS) using genetic scores for thyroid hormones as exposure variables and phecodes as the outcome variables. A PheWAS can be thought of as the inverse of a GWAS as it explores the association between a specific genetic variant (or GRS) and a wide range of phenotypes or clinical outcomes.^34^ We conducted logistic regression analyses to explore the associations between the GRS for TSH and FT4 and 1570 phecodes adjusting for age, sex, test centre, and the first ten genetic principal components. We included only outcomes with more than 200 cases in the current analysis. We report odds ratios or beta estimates for each 1 standard deviation unit increase in log-TSH or FT4 values.

To account for multiple testing, all statistical analysis were rigorously corrected for multiple testing using the Benjamini-Hochberg procedure controlling the false-discovery rate at 5%.

The GRS was calculated using Stata version 17.0. All other statistical analyses were conducted using R (version 4.0.3, R Foundation for Statistical Computing, Vienna, Austria).

#### Mendelian randomization

To further test for a homogenous dose–response, and hence likely causal relationship, between genetic differences in TSH and FT4 levels and the traits identified in the first step, we subsequently performed a two-sample Mendelian randomization analysis.

**Fig. 1:**
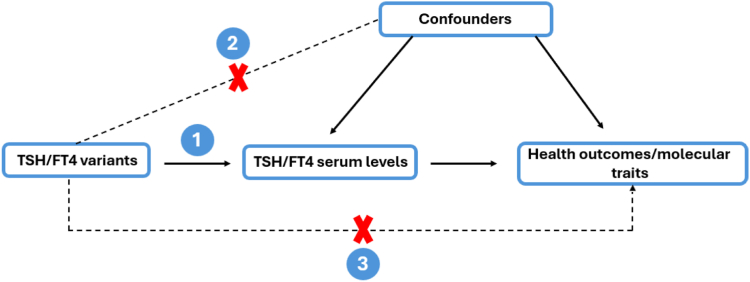
Conceptual framework for Mendelian randomization displaying the three assumptions that must hold for the results to be valid: (1) the genetic variant must be associated with the risk factor; (2) the genetic variant should not be associated with confounders; (3) the genetic variant should affect the outcome only through the risk factor.

For a Mendelian randomization analysis to be valid, three assumptions must hold (Fig. 1). First, the genetic variant must be associated with the risk factor (relevance).^35^^,^^36^ Second, the genetic variant is independent of the exposure-outcome association confounders (exchangeability).^35^^,^^36^ Third, the genetic variant is independent of the outcome, conditional on the exposure and confounders, i.e., there is no path from the genetic variant to the outcome that does not go through the exposure (exclusion restriction).^35^^,^^36^

Summary-level data for the identified metabolites were obtained from a recent GWAS for plasma metabolites.^33^ For the identified phecodes, summary-level statistics were obtained by running SNP–disease associations in 351,934 unrelated white European individuals. Two-sample MR was performed using the MR-PRESSO test in R.^37^ One of the fundamental assumptions of MR is the absence of horizontal pleiotropy, as its presence can lead to inaccurate causal estimates and possible false-positive causal associations.^37^ The MR-PRESSO test first detects if horizontal pleiotropy is present (the MR-PRESSO global test), and subsequently corrects for it by removing outliers.^37^

We further conducted a phenome-wide MR to assess the associations between genetic differences in TSH and FT4 levels and more than 2500 outcomes using the MRC Integrative Epidemiology Unit (IEU) OpenGWAS data infrastructure.^38^ We obtained the effect estimates for the SNPs associated with the included outcomes from GWAS summary statistics that are included in the MRC IEU OpenGWAS data infrastructure.^38^ If the selected instrumental variable (SNP) for TSH or FT4 was not available in the outcome GWAS statistics, it was replaced with a proxy SNP (r^2^ >0.8) which was obtained using the LDlink R package.^39^ Only outcomes that had 80% of the TSH or FT4 variants (or their proxies) available for the MR were included in the analyses.

#### Statistical colocalization analysis

For all autosomal variants outside of the extended MHC region associated with TSH and also associating at least suggestively (p < 10^−6^) with one of the traits highlighted in MR analysis, we implemented statistical colocalization.^40^ We downloaded matching summary statistics from the IEU OpenGWAS and defined 1 Mb window around the lead TSH signal (no such analysis was done for FT4 due to the absence of significant effects) and implemented colocalization using the R package coloc using default priors, apart from adjusting the prior for a shared signal to p_12_ = 5 × 10^−6^. We declared a posterior probability >80% for hypothesis 4 as evidence for a shared genetic signal.

#### Ethics

In the UKB, all participants gave informed written consent, and ethical approval was obtained from the North West Centre for Research Ethics Committee.^31^ This research has been conducted under the application 44448. All participants in the EPIC-Norfolk study gave informed written consent, and the study was approved by the Norwich Local Ethics Committee (previously known as Norwich District Ethics Committee; REC Ref: 98CN01). All participants gave informed written consent in the Fenland study, and the study was approved by the Cambridge Local Research Ethics Committee (NRES Committee—East of England, Cambridge Central, ref. 04/Q0108/19).

#### Role of funders

The funding source was not involved in the study design, in the collection, analysis, and interpretation of data, or in the writing of the report.

## Results

### Demographic characteristics

Mean age (± standard deviation) was 56.5 ± 8.1, 59.2 ± 9.3, and 48.6 ± 7.5 years for the UKB, EPIC-Norfolk, and Fenland study, respectively (Supplementary Table S2). Around 54% of the participants were female in all three studies.

### Associations between genetically predicted TSH and FT4 levels with plasma metabolites, proteins, and clinical biomarkers

We first investigated which molecular pathways may be affected by genetically predicted TSH and FT4 levels in >8000 participants of the Fenland and EPIC-Norfolk studies. We observed high specificity of the TSH and FT4 GRS being almost exclusively associated with directly related plasma measures among approximately 5000 proteins and 1000 metabolites tested (Supplemental Figure S1, and Supplemental Tables S3–S6). As expected, both the TSH and FT4 GRS were associated with thyroxine measured on the metabolomics platform (β: −0.148, adjusted p: 0.0002 and β: 0.417, adjusted p: 3.55 × 10^−21^, respectively) and TSH as measured by the proteomics assay (β: 0.681, adjusted p: 2.99 × 10^−104^). However, we identified only one non-thyroidal association. The FT4 GRS was significantly, but only moderately, inversely associated with plasma levels of sphingomyelin (beta coefficient [β]: −0.145, adjusted p: 0.036).

Having established the specificity of our instruments and to avoid false-negative findings due to the moderate sample sizes, we next tested for associations of genetically predicted TSH and FT4 levels with 325 ^1^H nuclear magnetic resonance spectroscopy (^1^H NMR) measures in the UKB^41^ (Supplementary Tables S9 and S10). After correction for multiple testing, only the TSH GRS was significantly inversely associated with plasma levels of tyrosine (Table 1; β = −0.043, adjusted p = 0.003), but no associations with the FT4 GRS passed this threshold and the associations were overall weak (smallest p-value = 0.49).

**Table 1:** Association between genetic predictors of thyrotropin (TRH) and UK biobank biochemistry markers and cardiovascular risk factors and ^1^H nuclear magnetic resonance (NMR) metabolomic biomarkers.

UKBB biomarkers	Genetic risk score			Mendelian randomization			
	Beta	SE	Adjusted p-value	Beta	SE	p-value	Outlier-corrected
SHBG	−0.060	0.005	9.37E-36	−0.046	0.009	1.33E-06	Yes
Testosterone	−0.028	0.003	3.51E-18	−0.024	0.005	2.11E-05	Yes
eGFR	−0.047	0.004	2.58E-28	−0.031	0.008	3.29E-04	Yes
Creatinine	0.042	0.004	6.88E-26	0.024	0.007	1.91E-03	Yes
Total cholesterol	0.030	0.005	4.06E-08	0.027	0.009	5.80E-03	Yes
NMR biomarkers							
Tyrosine^a^	−0.043	0.010	0.003	−0.039	0.011	3.72E-04	Yes

UKBB, UK Biobank; SE, standard error; SHBG, sex hormone binding globulin, eGFR, estimated glomerular filtration rate, NMR, nuclear magnetic resonance.

a Scaled.

In contrast, we identified associations between 18 out of the 33 clinical chemistry markers and the TSH GRS (Table 1, Supplemental Table S7) likely reflecting the greater power including 351,934 participants. This included inverse associations between the TSH GRS and markers of lipid metabolism (e.g., LDL-cholesterol), kidney (e.g., estimated glomerular filtration rate (eGFR)) and liver function (e.g., alanine aminotransferase). However, when testing more rigorously for a dose–response relationship, that is, ruling out disproportional contributions from single genetic variants, using a two-sample MR framework, only associations with sex hormone-binding globulin (SHBG), testosterone, eGFR, creatinine, and total cholesterol remained, largely reflecting known actions of thyroid hormones on intermediary metabolism^42^ (Table 1; number of SNPs included in the analyses = 82).

### Associations between genetically predicted TSH and FT4 levels and health outcomes in the UKB

**Fig. 2:**
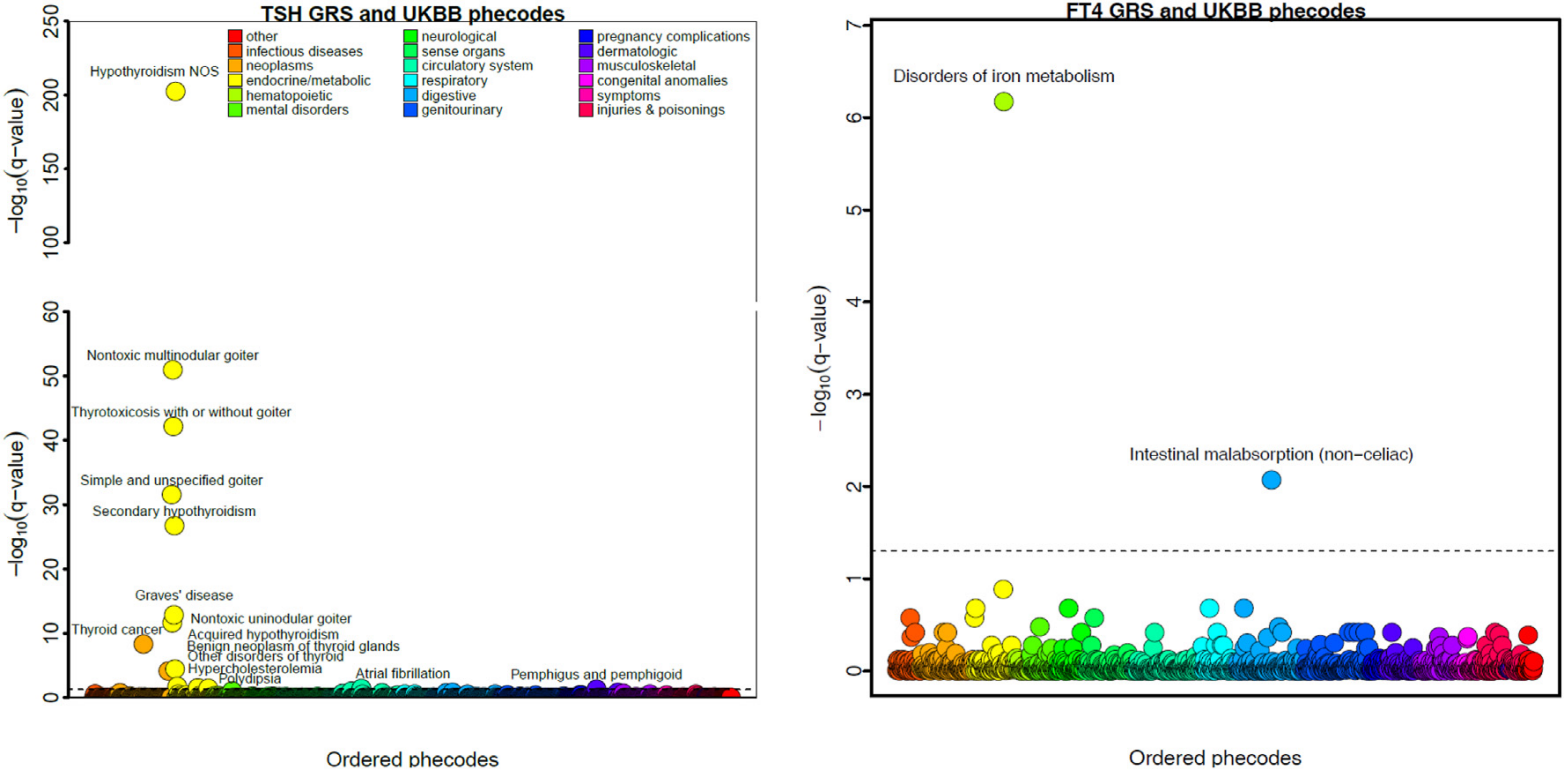
Phenome-wide association results for the genetic risk score (GRS) of thyrotropin (TSH) and free thyroxine (FT4) and UK Biobank phecodes. Logistic regression analyses were conducted to explore the associations between the GRS for TSH and FT4 and 1570 phecodes adjusting for age, sex, test centre, and the first ten genetic principal components. TSH, thyrotropin; FT4, free thyroxine.

We identified a total of 15 out of 1150 diseases to be significantly associated with the TSH GRS (Fig. 2, Table 2) that also persisted in more stringent two-sample MR analyses. Most of these outcomes were directly related to the thyroid gland, for example hypothyroidism, thyrotoxicosis, and thyroid cancer. Among the few non-thyroidal diseases, we replicated the known TSH GRS associations with atrial fibrillation (OR = 0.92, 95% confidence interval [CI]: 0.88–0.96, p-value = 3.61 × 10^−2^), and hypercholesterolemia (OR = 1.05, 95% CI: 1.02–1.08, p-value = 2.87E × 10^−2^),^18^ results that also held up when using the most recent GWAS (Supplementary Table S16).^43^^,^^44^ We further observed inverse associations with polydipsia (OR = 0.57, 95% CI: 0.42–0.78, p-value = 3.35 × 10^−2^), and pemphigus (OR = 0.51, 95% CI: 0.35–0.75, p-value = 4.52 × 10^−2^). We identified only one association for the FT4 GRS with intestinal malabsorption (OR = 1.55, 95% CI: 1.27–1.89, p-value = 8.48 × 10^−3^) that also persisted in the MR analyses.

**Table 2:** Association between genetic predictors of thyrotropin (TSH) and free thyroxine (FT4) and UK biobank phecodes.

Outcome	Genetic risk score				Mendelian randomization				
	OR	Lower CI	Higher CI	Adjusted p-value	OR	Lower CI	Higher CI	p-value	Outlier-corrected
TSH									
Hypothyroidism NOS	1.89	1.82	1.97	3.10E-203	1.71	1.60	1.83	7.27E-26	Yes
Nontoxic multinodular goiter	0.24	0.20	0.29	1.14E-51	0.25	0.17	0.36	7.44E-11	Yes
Thyrotoxicosis with or without goiter	0.57	0.52	0.61	6.78E-43	0.57	0.50	0.64	1.48E-14	Yes
Simple and unspecified goiter	0.34	0.29	0.41	2.72E-32	0.36	0.28	0.48	2.19E-10	Yes
Secondary hypothyroidism	0.47	0.41	0.53	1.85E-27	0.42	0.34	0.51	4.97E-13	Yes
Graves' disease	0.43	0.35	0.53	1.35E-13	0.46	0.35	0.59	3.69E-08	Yes
Nontoxic uninodular goiter	0.46	0.38	0.57	2.25E-12	0.48	0.38	0.60	1.58E-08	Yes
Thyroid cancer	0.49	0.39	0.60	4.49E-09	0.57	0.42	0.76	2.97E-04	Yes
Acquired hypothyroidism	2.06	1.56	2.72	3.48E-05	2.18	1.62	2.95	1.75E-06	No
Benign neoplasm of thyroid glands	0.49	0.37	0.65	6.87E-05	0.46	0.33	0.64	1.02E-05	No
Other disorders of thyroid	0.78	0.69	0.89	1.70E-02	0.81	0.67	0.98	3.05E-02	Yes
Hypercholesterolemia	1.05	1.02	1.08	2.87E-02	1.04	1.01	1.08	2.59E-02	Yes
Polydipsia	0.57	0.42	0.78	3.35E-02	0.61	0.44	0.86	5.78E-03	No
Atrial fibrillation	0.92	0.88	0.96	3.61E-02	0.92	0.87	0.97	2.13E-03	Yes
Pemphigus and pemphigoid	0.51	0.35	0.75	4.52E-02	0.50	0.34	0.72	3.50E-04	No
FT4									
Intestinal malabsorption	1.55	1.27	1.89	8.48E-03	1.68	1.31	2.15	2.75E-04	No

OR, odds ratio; CI, confidence interval; NOS, not otherwise specified.

### Phenome-wide Mendelian randomization

**Fig. 3:**
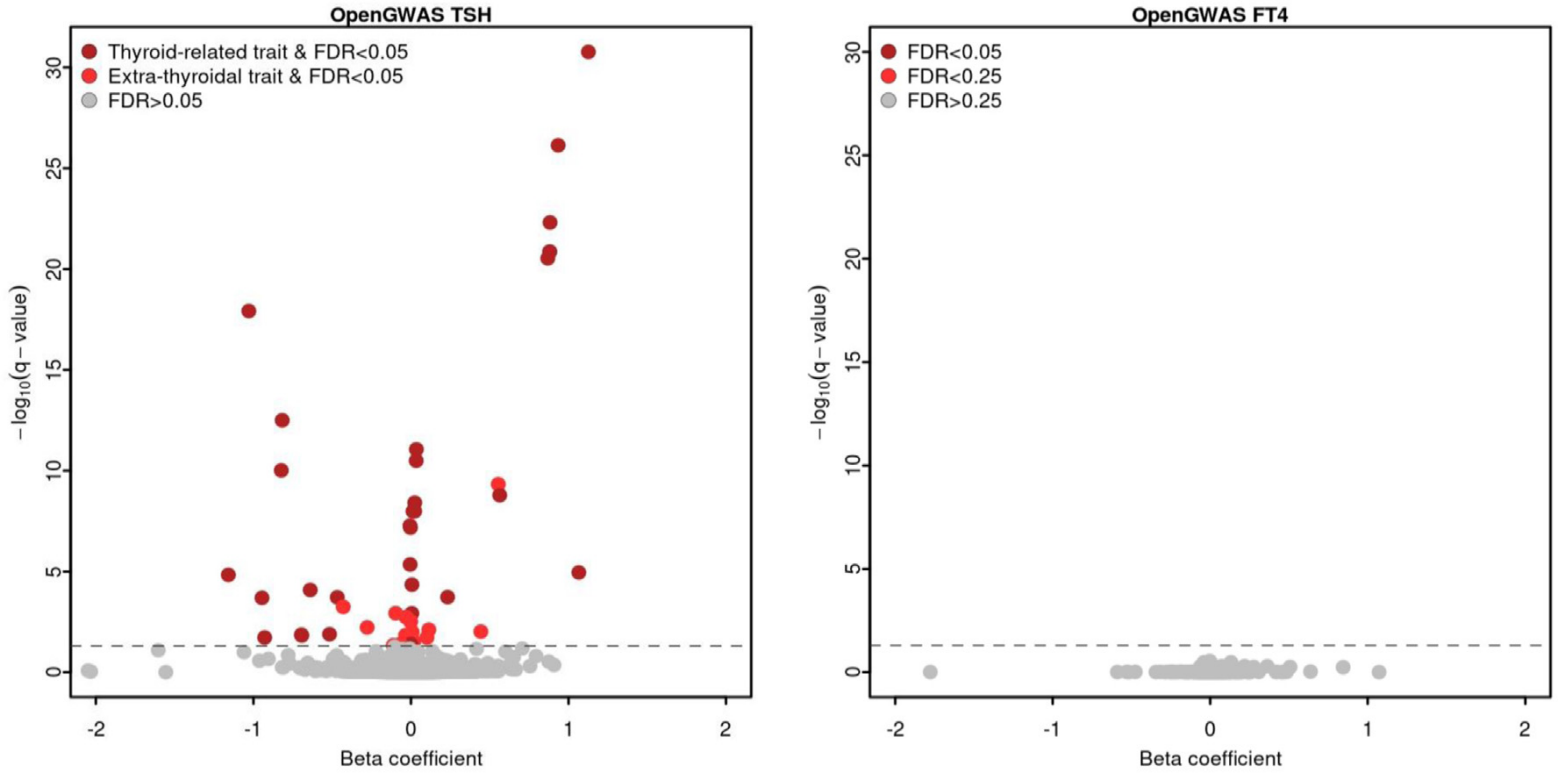
Phenome-wide Mendelian randomization results. The associations between genetic differences in TSH and FT4 level and more than 2500 outcomes were assessed using the MRC Integrative Epidemiology Unit (IEU) OpenGWAS data infrastructure. Effect estimates for the single nucleotide polymorphisms associated with the included outcomes were obtained from GWAS summary statistics that are included in the MRC IEU OpenGWAS data infrastructure. FDR, false discovery rate; TSH, thyrotropin; FT4, free thyroxine.

We finally performed phenome-wide MR using the OpenGWAS data infrastructure with genetically predicted TSH and FT4 as exposures to expand the spectrum of complex phenotypes, including endophenotypes like body fat distribution or lifestyle factors, and to increase statistical power for diseases less common in UKB.^38^ We identified 47 out of 5778 phenotypes to be significantly associated with the genetic liability to high but normal TSH levels in a two-sample MR setting (Supplementary Table S13, Fig. 3). Apart from replicating associations between TSH and thyroidal and non-thyroidal outcomes from our UKB analyses,^45^ we further identified associations with pulse rate (beta = −0.034, adjusted p-value = 0.014), non-alcoholic fatty liver disease (beta = 0.443, p-value = 0.010), unspecified lump in breast (beta = −0.278, p-value = 0.006), and depression medication (beta = 0.101, p-value = 0.019). After adjusting for multiple testing, we did not find significant associations between genetically predicted FT4 levels and any complex trait (Supplementary Table S14).

### Statistical colocalization

Finally, to explore whether some of the putative causal TSH/FT4—health characteristic associations may share molecular pathways, we performed statistical colocalization at all loci for which there was at least suggestive evidence for an association. We observed evidence at only six loci that TSH signals colocalized with phenotypes showing evidence for significant MR effect, demonstrating that our results point towards an effect of changes in TSH levels and subsequent shifts in thyroid hormone signalling rather than shared molecular causes (Supplementary Table S15).

## Discussion

In the present study, we systematically assessed the associations between genetically determined thyroid function within the normal range and a broad spectrum of metabolites, proteins, biomarkers, and health outcomes, and found little evidence of an association between genetic differences in thyroid function within the normal range and non-thyroidal phenotypes. We have found genetic evidence for causal associations for only very few, well-established, non-thyroidal diseases, most of which reflect the known biology of thyroid hormones, including lipid metabolism or pulse regulation. Our results suggest that previously reported associations between thyroid function parameters within the normal range and disease outcomes may have been affected by reverse causation or confounding.

In phenome-wide analysis, covering a broad range of molecular, intermediate as well as complex phenotypes, and diseases, we observed little evidence for a putatively causal role of genetically predicted variation of TSH and FT4 within the normal range. These findings contrast with the increasing number of non-genetic observational studies that point towards the pleiotropic effects of thyroid function (within the normal or subclinical range) on a wide variety of endpoints.^1^, ^2^, ^3^, ^4^, ^5^ Possible explanations include reverse causation, by which subclinical forms of diseases raise thyroid hormone levels, or missing or imperfect adjustment for common confounders. Examples of the latter are factors such as obesity that can impact TSH levels as well as increase the risk of cardiovascular and cancer endpoints, which can result in the identification of significant observational associations between thyroid hormones and disease endpoints. MR and genetic analysis can, to some extent, circumvent these problems, as alleles are randomly distributed at conception and do not change throughout life. Given the ready availability of genetic instruments and association statistics for numerous endpoints, it might be advisable to perform MR-type analysis as a necessary follow-up analysis to non-genetic observational studies.

It is currently recommended to screen for thyroid function in the presence of certain disease states, e.g., to establish the underlying cause of atrial fibrillation,^46^ among others, which is supported by our findings. Similarly, the association between thyroid function and total cholesterol is well-described in the literature and certain guidelines recommend screening for thyroid dysfunction in patients with newly diagnosed dyslipidemia.^47^ We found, however, little, if any, evidence, for strong links to non-thyroidal diseases beyond these established examples. Most of the biomarker links we identified are in line with the role of thyroid hormones in normal regulation and homeostasis, but without obvious disease consequences, including effects on sex-hormone metabolism.^48^

The large number of thyroid-related outcomes thought to be causally related to genetically predicted TSH levels might be explained by two distinct hypotheses. Firstly, in the last few decades, TSH receptors have been found to exist in extra-thyroidal tissues, possibly suggesting a direct role of TSH on peripheral organs.^49^ Secondly, TSH is the most sensitive screening test used to diagnose thyroid dysfunction (provided the absence of pituitary or hypothalamic disease).^9^ Therefore, people genetically predisposed to high/low levels of TSH that are close to diagnostic cut-offs might be diagnosed earlier, or more frequently, as compared to patients with levels genetically anchored in the middle of the reference ranges. Such a personal set point has already been widely discussed in the literature.^9^ For example, even though an individual might have a TSH level within the reference range for the general population, this TSH value might actually be pathological for this particular individual.^9^ Accounting for genetic contribution to TSH and/or FT4 levels may possibly help advance diagnosis and treatment of patients with borderline diagnostic values.

Our results are in line with previous studies that have shown that higher TSH levels may have a protective role on thyroid cancer risk.^19^^,^^50^ TSH is known to promote thyroid cancer growth^19^^,^^51^ and current guidelines recommend suppressing TSH in patients with intermediate- and high-risk thyroid cancer.^51^ However, it has been postulated that lower TSH levels can result in less differentiation of the thyroid epithelium, which, in consequence, can increase the risk of malignant transformation.^19^^,^^52^

While we systematically investigated the putative causal consequences of variation in thyroid function within the normal range, a number of limitations should be taken into account when interpreting our findings. Firstly, our study was conducted almost exclusively among people of European ancestry. Similar analyses in ethnically diverse populations, including those with historically different access to iodine, need to be carried out to test generalization of our results. Secondly, although our genetic instruments for thyroid function predominately included TSH and FT4 levels within the normal range, one of the cohorts from the TSH GWAS meta-analysis (the HUNT study) also potentially included individuals with subclinical thyroid dysfunction. However, a sensitivity analysis carried out within the HUNT study in which individuals with a TSH value outside the reference range were excluded (i.e., with subclinical thyroid dysfunction) revealed similar results to the main analysis. Thirdly, genetic variants associated with hypothyroidism or hyperthyroidism have been found to be in high linkage disequilibrium with variants associated with TSH or FT4 in the normal range.^20^ Thus, some of our ‘positive’ findings might be driven by genetic liability to overt thyroid disease rather than variations within the normal range. It is also challenging to translate our findings into quantitative estimates for use by clinicians. Lastly, although we used the MR-PRESSO test to identify and correct for potential horizontal pleiotropy, we cannot rule out the presence of bias due to unmeasured horizontal pleiotropy.

### Conclusions

In this systematic assessment of the associations between genetically determined thyroid function within the normal range and a broad spectrum of metabolites, proteins, biomarkers, and health outcomes, we found little evidence of an association between genetic differences in thyroid function within the normal range and non-thyroidal phenotypes. Putatively causal associations between TSH and extra-thyroid-related outcomes warrant further investigation to rule out reverse causation even in genetic studies, by which genetic instruments for diagnostic biomarkers may introduce artificial associations with diseases for which the biomarker is used as a diagnostic tool.

## Contributors

H.A., M.P., and C.L. designed the analysis. H.A. and M.P. drafted the manuscript. H.A. M.P., J.L., A.W., J.C.Z., and I.D.S. carried out or supported the data analysis. H.A. and M.P. had access and verified the data reported in the manuscript. All authors contributed to the interpretation of the results and critically reviewed the manuscript. All authors read and approved the final version of the manuscript.

## Data sharing statement

All individual level data is publicly available to bona fide researchers from the UK Biobank (https://www.ukbiobank.ac.uk/). Data access for the Fenland and EPIC studies can be requested by bona fide researchers for specified scientific purposes through a simple application process via the study websites below. Data will either be shared through an institutional data sharing agreement, or arrangements will be made for analyses to be conducted remotely without the necessity for data transfer. Source code used for analysis in this study is available on GitHub (https://github.com/hebaalwan/Thyroid_hormones_MR).

## Declaration of interests

All authors declare no conflict of interest.
