# Supplementary material for: Testing for a causal role of thyroid hormone measurements within the normal range on human metabolism and diseases: a systematic Mendelian randomization
Source: eBioMedicine. 2024 Aug 26;107:105306. doi: 10.1016/j.ebiom.2024.105306 (PMC11400601; doi:10.1016/j.ebiom.2024.105306)
Supplement: Supplementary Figure S1 [file mmc1.docx]

**Supplementary Figure 1. Association between the genetic risk score (GRS) of thyrotropin (TSH) and free thyroxine (FT4) with plasma metabolites and proteins**


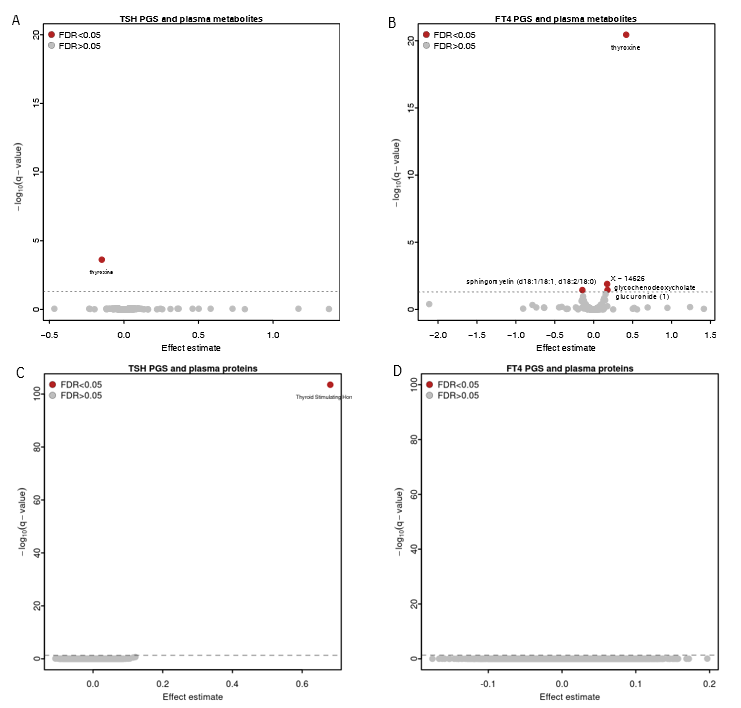


FDR: false discovery rate; TSH: thyrotropin; FT4: free thyroxine
